# Noninferiority and Safety of Nadolol vs Propranolol in Infants With Infantile Hemangioma: A Randomized Clinical Trial

**DOI:** 10.1001/jamapediatrics.2021.4565

**Published:** 2021-11-08

**Authors:** Elena Pope, Irene Lara-Corrales, Cathryn Sibbald, Carmen Liy-Wong, Nordau Kanigsberg, Beth Drolet, Jin Ma

**Affiliations:** 1 Division of Pediatric Dermatology, The Hospital for Sick Children, University of Toronto, Toronto, Ontario, Canada; 2 Division of Dermatology and Rheumatology, Children’s Hospital of Eastern Ontario, University of Ottawa, Ottawa, Ontario, Canada; 3 Department of Dermatology, University of Wisconsin–Madison; 4 Biostatistics Research Unit, University Health Network, Toronto, Ontario, Canada

## Abstract

**Importance:**

Propranolol for infantile hemangiomas (IH) has been shown to be effective and relatively safe. However, other less lipophilic β-blockers, such as nadolol, may be preferable in individuals who experience propranolol unresponsiveness or adverse events.

**Objective:**

To document the noninferiority and safety of oral nadolol compared with oral propranolol in infants with IH.

**Design, Setting, and Participants:**

This double-blind noninferiority prospective study with a noninferiority margin of 10% compared propranolol with nadolol in infants aged 1 to 6 months with problematic IH. The study was conducted in 2 academic pediatric dermatology centers in Canada between 2016 and 2020. Infants aged 1 to 6 months with a hemangioma greater than 1.5 cm on the face or 3 cm or greater on another body part causing or with potential to cause functional impairment or cosmetic disfigurement.

**Interventions:**

Oral propranolol and nadolol in escalating doses up to 2 mg/kg/d.

**Main Outcomes and Measure:**

Between-group differences comparing changes in the bulk (size and extent) and color of the IH at week 24 with baseline using a 100-mm visual analog scale.

**Results:**

The study included 71 patients. Of these, 36 were treated with propranolol. The mean (SD) age in this group was 3.1 (1.4) months, and 31 individuals (86%) were female. Thirty-five infants were treated with nadolol. The mean (SD) age in this group was 3.2 (1.6) months, and 26 individuals (74%) were female. The difference in IH between groups by *t* test was 8.8 (95% CI, 2.7-14.9) for size and 17.1 (95% CI, 7.2-30.0) for color in favor of the nadolol group, demonstrating that nadolol was noninferior to propranolol. Similar differences were noted at 52 weeks: 6.0 (95% CI, 1.9-10.1) and 10.1 (95% CI, 2.9-17.4) for size and color improvement, respectively. For each doubling of time unit (week), the coefficient of involution was 2.4 (95% CI, 0.5-4.4) higher with nadolol compared with propranolol. Safety data were similar between the 2 interventions.

**Conclusions and Relevance:**

Oral nadolol was noninferior to oral propranolol, indicating it may be an efficacious and safe alternative in cases of propranolol unresponsiveness or adverse events, or when faster involution is required.

**Trial Registration:**

ClinicalTrials.gov Identifier: NCT02505971

## Key Points

**Question:**

Is nadolol noninferior to propranolol in the management of infantile hemangioma?

**Findings:**

In this randomized clinical trial, infantile hemangiomas treated with nadolol showed a larger change in size and color compared with propranolol, demonstrating that nadolol was noninferior to propranolol.

**Meaning:**

Nadolol may be an efficacious and safe alternative to propranolol in the treatment of infants with infantile hemangiomas.

## Teaser

This randomized clinical trial evaluates the noninferiority and safety of oral nadolol compared with oral propranolol in infants with infantile hemangiomas.

## Introduction

Infantile hemangiomas (IHs) are the most common tumors in infancy and affect 4.5% of infants worldwide.^1,2^ They are benign vascular endothelial neoplasms, seen more frequently in female infants (5:1 ratio), in premature infants with a birth weight of less than 1500 g, and in twins. The indications for treatment are limitation or interference with normal functions (eg, breathing, vision, and hearing), persistent or intractable ulceration, and permanent structural abnormalities resulting in cosmetically unacceptable deformities.^3^ In June 2008, spurred by an anecdotal observation, the β-blocker propranolol was reported to be beneficial in the treatment of IHs either alone or in combination with corticosteroids.^4^ A large international randomized clinical trial documented the efficacy and relative safety of propranolol in patients with IHs.^5^

While the efficacy data are strong and favorable, the ability of propranolol to cross the blood-brain barrier with potential unproven long-term neurocognitive effects, as well as the higher pharmacokinetic variability of propranolol,^6^ prompted our group to evaluate other β-blockers in the treatment of IHs.^7^ Nadolol is a synthetic nonselective β-blocker with no known intrinsic sympathomimetic or membrane-stabilizing activity.^8,9,10^ It is currently used in the pediatric age group in the treatment of hypertension,^11^ rhythm abnormalities (eg, atrial tachyarrhythmias and ventricular arrhythmias), and overt aggression in individuals with developmental delays.^12^ The usual dose for cardiovascular problems is 1 to 4 mg/kg/d orally.^9^ Unlike propranolol, nadolol is not metabolized by the liver and is excreted unchanged, primarily by the kidneys and in a lesser amount through the feces. The half-life of nadolol is 12 to 24 hours, reaching steady-state serum concentrations in 6 to 9 days with a once-daily dosage.^9^ Nadolol has no intrinsic sympathomimetic activity and, in contrast to propranolol, has little myocardial depressant activity and does not have an anesthetic-like membrane-stabilizing action.^13^ In addition, nadolol is less lipophilic and therefore does not cross the blood-brain barrier, which decreases the risk of adverse events in the central nervous system, such as sleep disturbance, behavioral changes, and effects on memory.^7,9^

We previously published a pilot study to examine the efficacy and safety of nadolol through comparison with a historical cohort treated with oral propranolol. At similar mean dosages (2.1 mg/kg/d), 10 patients receiving nadolol had a mean (SD) IH involution of 51% (18.5%) at the 4-week visit, 83% (13.9%) at the 12-week visit, and 97% (3.5%) at the end of the study (24 weeks). In contrast, 10 patients receiving propranolol had a mean (SD) IH involution of 28% (10.4%), 56% (16.6%), and 86% (14.8%) at 4, 12, and 24 weeks, respectively (*P* < .001).^14^ Nadolol was well tolerated with no significant adverse events.

The preliminary data supported our hypothesis that nadolol is an efficacious and, given its pharmacological properties, alternative modality to propranolol for use in the treatment of IHs. The objective of this study was to document the noninferiority and safety of oral nadolol compared with oral propranolol in the treatment of IHs.

## Methods

The study was conducted between 2016 and 2020 at 2 tertiary academic pediatric dermatology clinics: the Hospital for Sick Children in Toronto, Ontario, Canada, and Children’s Hospital of Eastern Ontario, Ottawa, Ontario, Canada. The study was approved by the ethics boards at each institution, and families provided written informed consent. Infants with a corrected gestational age of 1 to 6 months were included if they had hemangioma greater than 1.5 cm on the face or 3 cm or greater on another body part either causing or with the potential to cause functional impairment (eg, amblyogenic IH or ulcerated hemangioma) or causing or having the potential to cause cosmetic disfigurement (eg, nasal tip, glabella location). The exclusion criteria were contraindications to β-blockers (eg, hypotension, bradycardia, hypoglycemia, cardiac disease associated with decreased ejection fraction or second-degree or greater heart block, bronchospasm, and allergic rhinitis); corrected gestational age less than 1 month at screening; posterior fossa abnormality, hemangioma, arterial cerebral anomalies, cardiac anomalies, eye anomalies, sternal defects, or cerebral arteriopathy at risk of stroke; patients or breastfeeding mothers were receiving treatment with antiarrhythmic agents, calcium channel blockers, angiotensin-converting enzyme inhibitors, inotropic agents, vasodilators, hypoglycemic agents, neuroleptics, antiacids, benzodiazepines, thyroxine, or warfarin; patients were being treated with an oral β-blocker or other agent (eg, systemic steroids and vincristine) within 2 weeks from randomization; patients were being treated with topical timolol within 1 week prior to randomization; and presence of vascular tumors other than infantile hemangioma (eg, pyogenic granuloma and hemangioendothelioma).

Investigators and parents of patients were blinded to the intervention. To prevent unintentional unblinding, the medication was dispensed in brown bags by the research pharmacy. Patients were randomized by the research pharmacist in blocks of 4 to either nadolol (oral suspension, 10 mg/mL) or propranolol (oral suspension, 5 mg/mL). The medication was started at 0.5 mg/kg/d twice a day and titrated weekly by 0.5 mg/kg/d until the maximum dose of 2 mg/kg/d was reached. In all subsequent visits up to week 24, the dosage was adjusted based on clinical response and patient weight.

The blinded duration of the study was 24 weeks, after which parents were given the option to continue on the randomization intervention or to change interventions. The follow-up visits after week 25 occurred every 2 months until week 52 and were aimed at monitoring further clinical responses and adverse events. If there was complete response or no evidence of further improvement for 4 weeks, a weaning schedule (10% decrease of the dose per month) was implemented. Complete response was defined as complete size involution or minimal residual epidermal changes (telangiectasias, anetoderma, and gray-red discoloration), whereas partial response was defined as a 50% to 75% decrease in the size, extent, and coloration.

All visits involved monitoring of vital signs, standardized photography (front view and side view with color checker calibration card), height and weight measurements, and complete history and physical examinations. In addition, hemangioma activity scale^15^ and hemangioma severity scale^16^ were calculated. For the visits that involved planned dose escalation, the medication was administered in the clinic, followed by monitoring of vital signs and blood glucose level at 1 to 2 hours after administration. Adverse events were reviewed and documented at each visit.

### Outcome Measures

IHs are 3-dimensional lesions with variable extent, depth of involvement (superficial, deep, or mixed), and color. As such, it is difficult to adequately quantify the size of 1 particular lesion and to monitor proliferation or involution over time. Furthermore, IHs occur at a time when the human body grows rapidly. These factors limit a universal, standardized way of measuring IH over time or with treatment and to date, to our knowledge, there are no objective tools. A visual analog scale (VAS) was used to quantify changes in the visible bulk (size and extent). The VAS consisted of a 100-mm scale with 3 anchors (−100 indicates ongoing proliferation; 0, no change; and +100, complete shrinkage of the tumor), where 5 mm represents a 10% change in either direction. Similarly, a VAS for color changes was used with 3 anchors (−100 indicates persistent redness or violaceous discoloration; 0, no change; and +100, normal skin color). Both size and color changes were determined by comparing clinical photographs at various time points. This version of the VAS has been used in several other studies, showing great interperson reliability (intraclass correlation coefficient [ICC], 0.87; *P* < .001) and responsiveness to change.^14,17^

The primary efficacy criterion was between-group differences in the bulk and color changes of the IH at week 24 compared with baseline using VAS scores. Efficacy secondary outcome measures were percent change in IH bulk using VAS scores at 4, 12, and 52 weeks, time and dose required to reach 75% and 100% tumor shrinkage, interrater reliability of the VAS scores, percentage of patients achieving functional correction (eg, normal vision) at 24 and 52 weeks, and percentage of patients with residual changes (ie, telangiectasias, discoloration, fibro-fatty changes, and anetoderma). Safety outcome measures consisted of determining frequencies of adverse events observed (eg, drop in heart rate and blood pressure of more than 10% from baseline or less than one-third percentile for age and low blood glucose level) and all reported adverse events.

### Power Calculation and Statistical Methods

We estimated that 39 patients per group ensured that the study had 80% power to determine that nadolol was noninferior to propranolol at a noninferior margin of 10%, a significance level of 2.5%, based on the assumption that standard deviation in the overall improvement as measured by change from baseline is 15% for each of the groups (as seen in our pilot study).^14^

Descriptive statistics were used for patient demographic and baseline data, which were summarized by frequencies and proportions for categorical variables and means and SDs for continuous variables. The 2-sample *t* tests and the χ^2^ test were used for continuous variables and categorical variables from primary and secondary outcomes as appropriate at weeks 24 and 52. The *t* test was used to assess noninferiority in responses between the 2 groups with 95% CIs around the difference. Fisher exact test was used for the categorical safety data. The difference between 2 groups at each time point was visualized with box plots. Visual correction of IH color was performed using a histogram. To assess the difference of mean VAS score between 2 groups for weeks 4, 12, 24, and 52, a mixed model with a base-2 log scale of time and interaction between time and treatment was fitted. The interaction coefficient was the difference between groups in the trend, where the trend was a log-week increase. Cox proportional hazards models were used to determine the association between the medicine and time to achieve 75% and 100% tumor shrinkage. Kaplan-Meier curves were generated to visualize the time to 75% and 100% tumor shrinkage probabilities in each treatment group and compared using log-rank test. *P* values were 2-tailed with a significance threshold of .05. Analyses for the blinded portion were as intention to treat. Statistical analyses were performed using R version 4.0.3 (the R Foundation).

## Results

**Figure 1.**
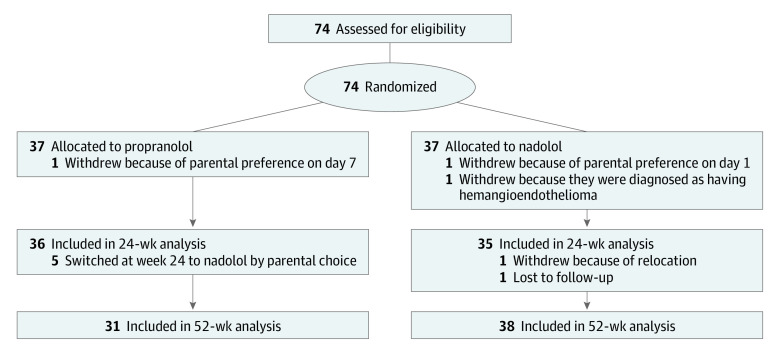
CONSORT Diagram

The cohort included 74 patients, randomized equally to 1 of the 2 interventions (Figure 1). Three patients withdrew from the study within 1 week of enrollment and were not included in the final analysis. The groups were equally distributed in terms of demographic and clinical characteristics (Table 1). The study was stopped early (74 instead of 78 patients) owing to slow enrollment during the COVID-19 pandemic.

**Table 1.** Demographic and Clinical Baseline Data

Characteristic	No. (%)	
	Nadolol (n = 35)	Propranolol (n = 36)
Age at enrollment, mean (SD), mo	3.2 (1.6)	3.1 (1.4)
Female	26 (74)	31 (86)
Male	9 (26)	31 (14)
Term pregnancy	32 (91)	26 (72)
Multiple gestations	3 (9)	3 (8)
Past medical history	3 (9)^a^	4 (11)^b^
Medication history	9 (26)^c^	20 (56)^d^
Infantile hemangioma subtype		
Superficial	0	2 (6)
Deep	3 (9)	8 (22)
Mixed	32 (91)	26 (72)
Infantile hemangioma morphology		
Localized	30 (86)	31 (86)
Segmental	4 (11)	3 (8)
Indeterminate	1 (3)	2 (6)
Multiple infantile hemangiomas	12 (34)	6 (17)
Head and neck location	31 (89)	33 (92)
Hemangioma scale, mean (SD)		
Severity	10.6 (3.4)	10.61 (3.3)
Activity	8.59 (1.8)	9.58 (1.4)
Complications of hemangioma	14 (40)	14 (38.9)
Visual obstruction	3 (21)	2 (14)
Feeding problems	1 (7)	3 (21)
Ulceration	8 (57)	8 (57)
Distortion of landmarks	1 (7)	1 (7)
Nasal obstruction	1 (7)	0

^a^ Hypoglycemia (3).

^b^ PDA (1), cleft palate (1), benign heart murmur (1), umbilical hernia (1).

^c^ Vitamins.

^d^ Vitamins (17), antibiotic (1), iron (1), probiotic (1).

### Efficacy Data

The 24-week difference between groups by *t* test was 8.8 (95% CI, 2.7-14.9) for size and 17.1 (95% CI, 7.2-30.0) for color in favor of nadolol. Similarly, at 52 weeks, the difference between groups was 6.0 (95% CI, 1.9-10.1) and 10.1 (95% CI, 2.9-17.4) for size and color improvement, respectively (Table 2). The mean (SD) size involution compared with baseline was 97.9% (3.7%) in the nadolol group vs 89.1% (17.7%) in the propranolol group, similar to mean (SD) color fading, which was 94.5% (8.7%) in the nadolol group vs 80.5% (17.9%) in the propranolol group (eFigures 1 and 2 in Supplement 2). The mean (SD) hemangioma activity scale at 24 weeks was 1.6 (1.7) in the nadolol group vs 3.2 (3.2) in the propranolol group (*P* = .002) and 0.6 (0.8) vs 1.6 (1.4) at 52 weeks (*P* = .001) (eTable in Supplement 2).

**Table 2.** Primary Outcome Measure: Difference in Responses Comparing Nadolol With Propranolol

Assessment wk^a^	Difference (95% CI)		
	Size	Color	Overall
1	−2.0 (−9.7 to 5.7)	2.7 (−3.8 to 9.2)	−1.8 (−8.7 to 5.1)
2	−1.9 (−9.8 to 7.3)	1.85 (−6.2 to 9.9)	−1.9 (−9.6 to 3.9)
3	−2.5 (−11.2 to 6.1)	0.1 (−9.4 to 9.6)	−3.2 (−11.4 to 5)
4	0.1 (−8.2 to 8.3)	1.4 (−7.3 to 10.2)	−1.4 (−9.4 to 6.6)
8	3.9 (−3.8 to 11.6)	3.4 (−6.2 to 13.1)	1.8 (−5.9 to 9.6)
12	7.9 (0.9 to 15.0)	12.1 (2.6 to 21.6)	7.9 (0.6 to 15.2)
16	6.9 (−0.2 to 14.1)	13.7 (5.1 to 22.3)	8.4 (2.1 to 14.8)
20	8.7 (3.1 to 14.4)	15.4 (6.7 to 24.2)	10.3 (4.0 to 16.6)
24	8.8 (2.7 to 14.9)	17.1 (7.2 to 30.0)	9.8 (3.9 to 15.6)
32	8.9 (2.1 to 15.8)	9.9 (2.3 to 17.5)	7.8 (1.7 to 13.9)
40	6.1 (1.7 to 10.5)	10.64 (2.6 to 18.7)	6.8 (1.5 to 12.1)
52	6.0 (1.9 to 10.1)	10.1 (2.9 to 17.4)	6.64 (1.9 to 11.4)

^a^ Each assessment was done at each time interval compared with baseline.

**Figure 2.**
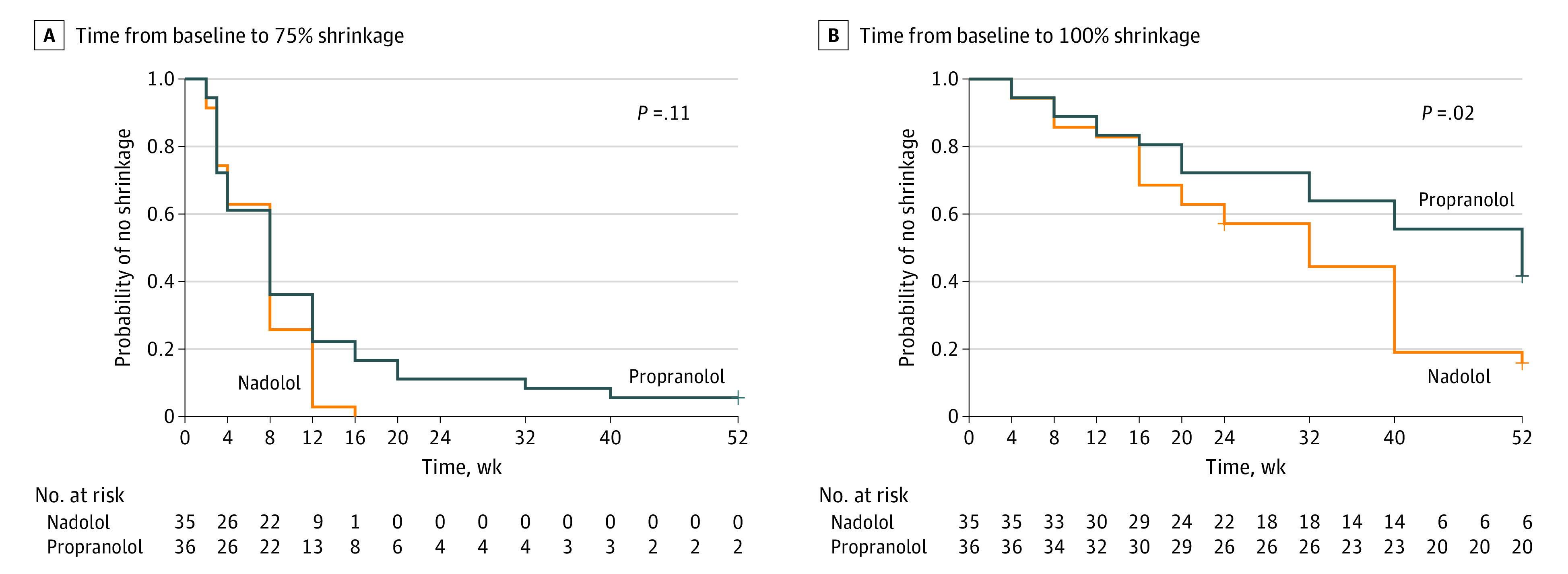
Kaplan-Meier Plot

More patients in the nadolol group achieved 75% and 100% involution compared with those in the propranolol group (Figure 2; eTable in Supplement 2). In the mixed-model analysis, for each doubling of unit of time (week), the coefficient of involution was 2.4 times higher (95% CI, 0.45-4.4) for nadolol compared with propranolol. Univariable Cox proportional hazards models showed that nadolol had a 59% higher rate in achieving 75% shrinkage of IH compared with propranolol (hazard ratio, 1.6; 95% CI, 0.9-2.6; *P* = .07) and 105% higher in achieving 100% shrinkage (HR, 2.1; 95% CI, 1.2-3.7; *P* = .02). Kaplan-Meier analysis (Figure 2) confirmed that nadolol was faster than propranolol either in achieving 75% shrinkage (log-rank *P* = .11) or in achieving 100% shrinkage (log-rank *P* = .02). The ICC between the assessors showed good agreement for size 0.7 (95% CI, 0.6-0.8) but less so for color 0.5 (95% CI, 0.3-0.6). Five patients allocated to propranolol switched to nadolol after 24 weeks at parental request (4 because of perceived inadequate response and 1 because of severe nightmares) (eFigure 1 in Supplement 2).

### Safety Data

Both medications were well tolerated with expected adverse events (Table 3). Most patients experienced at least 1 adverse event (77.1% vs 94.4% at 0 to 24 weeks and 84.2% vs 74.2% at 24 to 52 weeks in the nadolol group and propranolol group, respectively). The median (IQR) number of reported adverse events was 2 (1-3) in the nadolol group vs 2 (1.8-3.3) in the propranolol group (*P* = .12). Most events were respiratory in nature, 77.8% (0 to 24 weeks) and 75.0% (24 to 52 weeks) in the nadolol group, comparable with 70.6% (0 to 24 weeks) and 78.3% (24 to 52 weeks) in the nadolol group, and not attributed to the medication use. One patient in the propranolol group experienced a grade 1 serious adverse event consisting of pneumonitis that required holding medication and admission to the hospital; the event ended with full recovery and no subsequent events after medication restart. Sleeping difficulties were the second most common adverse events, affecting 44.4% of patients in the nadolol group and 44.1% patients in the propranolol group at 0 to 24 weeks, with numbers decreasing significantly after 24 weeks (9.4% vs 4.3%) (Table 3). The most common sleep disturbance was an increase in awake nighttime episodes (50.0% in the nadolol group vs 66.7% in the propranolol group), with less frequency of nightmares and excessive crying (33.3% vs 33.3%), although most sleeping difficulties were thought by parents to be related to the medication use (50.0% in the nadolol group vs 80.0% in the propranolol group). One patient in the propranolol group had severe episodes of nightmares that did not recur after switching to nadolol.

**Table 3.** Safety Data^a^

Period	0-24 wk			25-52 wk		
Characteristic	No. (%)		*P* value	No. (%)		*P* value
	Nadolol (n = 35)	Propranolol (n = 36)		Nadolol (n = 38)	Propranolol (n = 31)	
Presence of AEs	27 (77)	34 (94)	.08	32 (84)	23 (74)	.47
Respiratory AEs						
Overall	21 (78)	24 (71)	.73	24 (75)	18 (78)	>.99
URTI	20 (95)	24 (100)	.95	21 (88)	18 (100)	.34
Bronchiolitis	1 (5)	2 (8)	>.99	3 (13)	0	.22
Pneumonia	0	0	>.99	1 (4)	2 (11)	>.99
No. of episodes			.68			.25
1	10 (48)	10 (48)		17 (71)	13 (68)	
2	8 (38)	6 (26)		5 (21)	4 (21)	
3	2 (10)	5 (22)		0	2 (11)	
4	1 (5)	1 (4)		0	0	
Gastrointestinal AEs						
Overall	7 (26)	15 (44)	.23	6 (19)	3 (13)	.85
Constipation	1 (14)	4 (27)	.89	1 (17)	1 (33)	.69
Diarrhea	3 (43)	6 (40)		4 (67)	2 (67)	
Vomiting	2 (29)	4 (27)		0	0	
Other	1 (14)	1 (7)		1 (17)	0	
Sleeping difficulties						
Overall	12 (44)	15 (44)	>.99	3 (9)	1 (4)	.86
Excessive crying, nightmares	4 (33)	5 (33)	.25	1 (33)	1 (100)	>.99
Increased frequency of awaked episodes	6 (50)	10 (67)		2 (67)	0	
Not classified	2 (17)	0		0	0	
Related to medication	6 (50)	12 (80)	.09	2 (67)	1 (100)	>.99
Behavioral changes	5 (19)	3 (9)	.46	0	0	>.99
Cold hands and feet	7 (26)	4 (12)	.27	0	0	>.99
Skin AEs						
Overall	2 (7)	5 (15)	.63	5 (16)	4 (17)	>.99
Eczema	1 (50)	1 (20)	.35	1 (20)	0	.16
Viral exanthems	0	3 (60)		4 (80)	2 (50)	
Bacterial infections	1 (50)	1 (20)		0	2 (50)	
Other AEs	10 (37)	12 (35)	>.99	5 (16)	5 (22)	.82
Serious AEs	0	0	NA	0	1 (0)^b^	.35

Abbreviations: AE, adverse events; NA, not applicable; URTI, upper respiratory tract infections.

^a^ Percentages for each column use the overall number as the denominator for each category.

^b^ Admission to hospital for pneumonia, fully recovered, not suspected to be related to the medication.

## Discussion

In this double-blinded randomized comparison trial of infants with problematic IHs, we found that oral nadolol was noninferior to oral propranolol at the same doses and had a similar safety profile. The efficacy of propranolol is well established, but with a wide-reported range in the magnitude of the response, dependent on the study design, severity of the IHs, and outcome method used. Our data demonstrated a mean (SD) decrease in size and extent of 97.9% (3.7%) vs 89.1% (17.7%) in the nadolol group vs the propranolol group at 24 weeks (eTable Supplement 2). Moreover, nadolol was 59% faster in achieving 75% shrinkage of IH compared with propranolol (*P* = .02) and 105% faster in achieving 100% shrinkage (*P* = .07) during the study period. In what was, to our knowledge, the seminal article and the largest randomized clinical trial on this topic to date,^5^ the primary efficacy measure was complete and near complete resolution of the IHs seen in 60% of patients treated with 3 mg/kg/d of propranolol for 6 months. Moreover, that particular cohort included only patients with nonproblematic IHs owing to the placebo arm. Baselga et al^18^ addressed this limitation by conducting a small open-label study (43 patients) using 3 mg/kg/d of propranolol in patients with problematic IHs. They reported 47% and 76% resolution (disappearance with minimal sequelae of color, texture, and bulk) of the IHs at 6 months and 12 months, respectively.^16^

Another difficulty in comparing current data with existing literature is the dosage used. While prior studies used higher doses, in practice, 2 mg/kg/d is preferred as showing good response and less frequency of adverse events.^19^ In studies summarizing efficacy data with 2 mg/kg/d, excellent and good response rates were similar to our data.^19,20^ The variability in the improvement assessment in the current literature underscores the difficulty of evaluating a response in a tridimensional lesion, with and without a superficial component, with initial darkening followed by lightening over the ensuing 6 to 12 months. In our study, we used separate VASs for size and color, recognizing that the process of size and color involution may not necessarily be linear and congruent in each given lesion. We found very good agreement between 2 independent investigators in terms of changes in size and extent (ICC, 0.73; 95% CI, 0.63-0.81) but less so for color (ICC, 0.48; 95% CI, 0.29-0.60). We believe that the discrepancy in color assessments, despite standardization for position and distance to the patient, were associated with other parameters that are hard to control from visit to visit (eg, room light and ambient temperature). Despite that, the color histogram seen in eFigure 2 in Supplement 2 provides a pictorial representation that may be useful for clinicians when providing counseling to the families. We believe that in the absence of a criterion standard, separate VASs for size and color may provide a more objective tool in the setting of a clinical trial rather than using binary or other categorical arbitrary scales.

There were no significant concerning safety signals in this study and no differences in reported adverse events between the 2 interventions. A considerable portion of patients experienced at least 1 mild adverse event (77.1% vs 94.4% at 0 to 24 weeks and 84.2% vs 74.2% at 24 to 52 weeks in the nadolol group vs the propranolol group, respectively), with a median of 2 in each intervention group. While these figures are high, they are similar to data from previous clinical trials (88.5% in the intervention group vs 67.8% in the placebo group).^21^ None of the patients experienced clinically significant or symptomatic hemodynamic changes or hypoglycemia after dose adjustments. Most of the events were respiratory in nature (more than 70%) and not attributed to the intervention. One patient treated with propranolol experienced a serious adverse event (pneumonitis that required admission with full recovery). Sleeping difficulties were the second most common adverse events (44.4% vs 44.1% at 0 to 24 weeks and 9.3% vs 4.3% at 24 to 52 weeks in the nadolol group vs the propranolol group, respectively). There are several important points about this association.^22^ More patients than previously reported experienced sleeping difficulties (ie, 9.3%,^23^ 19%,^19^ 3.7%,^24^ and 15.7%^25^), even when reporting data of the entire cohort: 34.3% vs 41.6% at 0 to 24 weeks and 7.8 vs 3.2% at 24 to 52 weeks in the nadolol group vs the propranolol group, respectively. This could be owing to increased awareness about this association and more stringent monitoring. Of reports of sleeping problems, 50% to 80% were attributed by parents to the medication, with 1 parent requesting changing medications after 24 weeks. On the other hand, a smaller percentage of patients experienced severe sleep disturbance (nightmares and excessive crying episodes). Another interesting observation is that sleeping difficulties decreased after 6 months, likely multifactorial in nature (normal maturation and development of more stable sleep patterns, decreasing the need to wake infants up for feeding as a prevention for hypoglycemia, and less exposure to medication owing to progressive weaning).

### Limitations

Our study had several limitations. Although VASs have been used in several cohort studies, they remain a subjective outcome measure. Recruitment was stopped short of 5 patients for feasibility reasons. However, given our results, further recruitment would have unlikely affected the results. Randomization was not done according to the hemangioma subtype, which may have introduced a response bias.

## Conclusions

In this trial, oral nadolol was noninferior to propranolol in the treatment of IHs. The efficacy data coupled with a more predictable pharmacokinetic profile and lower chance of crossing the blood-brain barrier may make nadolol a favorable alternative intervention in patients with IHs. Although nadolol demonstrated a faster and better response in patients with problematic IHs, further studies are needed to prove superiority over propranolol.

## Notes

Supplement 1. Trial protocol

Supplement 2. eTable. Secondary efficacy outcome measures eFigure 1. Efficacy data boxplots eFigure 2. Visual correction of IH color chart

Supplement 3. Data sharing statement
