# Supplementary material for: Noninferiority and Safety of Nadolol vs Propranolol in Infants With Infantile Hemangioma: A Randomized Clinical Trial
Source: JAMA Pediatr. 2021 Nov 8;176(1):1–8. doi: 10.1001/jamapediatrics.2021.4565 (PMC8576629; doi:10.1001/jamapediatrics.2021.4565)
Supplement: Supplement 1. — Trial protocol [file jamapediatr-e214565-s001.pdf]

PROTOCOL

Title

Nadolol versus Propranolol in Children with Infantile Hemangioma: A Randomized, Controlled, Double-blinded, Non-inferiority Trial

Qualified/Principal Investigator: Dr. Elena Pope
Co-/Sub-Investigators: Dr. Irene Lara-Corrales
Dr. Beth Drolet

Rationale

Worldwide, most patients with infantile hemangiomas (IH) that require systemic treatment are receiving oral propranolol despite the lack of strong evidence from large prospective studies. Propranolol crosses the blood brain barrier. The central nervous system blockade of the adrenergic system by propranolol may lead to short and long-term effects particularly if given long-term in patients during their early developmental stages.
We have accumulated preliminary evidence that nadolol, another non-selective beta-blocker, may be more efficacious than propranolol and safer due to less cardiodepressant effect and its inability to cross the blood brain barrier.
Considering the increasing number of patients under age of two that are currently treated with oral propranolol it is imperative to find alternate agents that are as efficacious and safer than propranolol. This randomized controlled trial will compare the efficacy and safety of nadolol and propranolol in a, prospective, non-inferiority study.

Background

Infantile Hemangiomas (IH) are the most common tumors of infancy and affect approximately 5-10% of Caucasian children. [1,2]They are benign vascular endothelial neoplasms seen more frequently in females (5:1 ratio), in premature babies with a birth weight of less than 1500 gm and in twins.
The natural history of IH consists of proliferation of the vascular endothelium and is clinically characterized by rapid increase in the size of the lesion, a bright red color of the lesion and increased local temperature. In most patients, the proliferative phase may span over the first year of life and is followed by slow involution over the next 5-10 years.
The undisputable indications for treatment are: limitation/interference with normal functions (breathing, vision, hearing, eg, etc.), significant disfigurement, persistent/intractable ulceration, bleeding, coagulation abnormalities, heart failure secondary to increased blood flow, and permanent structural abnormalities resulting in cosmetically unacceptable deformities. [3] More debatable indications are smaller facial lesions that will resolve over time, large lesions on other body parts with low risk of complications, etc. Despite location, most parents seek some form of therapy in the hope of controlling the growth or improving the appearance.

In June 2008, spurred by an anecdotal observation, the beta-blocker propranolol was reported to be beneficial in the treatment of IHs either alone or in combination with corticosteroids.[4] A large, worldwide RCT is in the final stages of completion, but smaller size studies have documented the efficacy and relative safety of propranolol in patients with IH. Propranolol is a non-selective beta-adrenergic agent. Upon oral administration, 60-70% of the drug is absorbed and metabolized through first pass in the liver, leaving 30-40% bioavailable,

with a half-life of 4 hours. [5] Due to its lipophilic properties propranolol easily penetrates blood brain barrier with CSF levels closely reflecting free plasma concentrations. [6] Although not exactly clear, the therapeutic effects of propranolol on IHs could be explained by vasoconstriction (immediately visible as a change in color, associated with a palpable softening of the hemangioma), decreased expression of vascular endothelial growth factor (VEGF) and basic fibroblast growth factor (bFGF) genes through down regulation of RAF – mitogen activated protein kinase pathway, and apoptosis of capillary endothelial cells. [7] In a recent systematic review, the response rate, defined as *any* improvement in the IH, ranged from 82-100%, with rebound seen in 17% of cases with propranolol given at doses of 2 mg/kg/day divided BID/TID. [8]

While all the preliminary data suggest that propranolol is efficacious, there are some limitations and restrictions to its widespread use; necessity for three a day dosing due to its short half life of 3-4 hours, unknown short and long-term cardiac effects and potential deleterious effects on the brain due to central beta-blockade.

The frequency of reported adverse events in the same systematic review of 1175 patients were sleep disturbance (including nightmares) in 3.7%, followed by asymptomatic hypotension- 2.8%, somnolence-2.2% and pulmonary symptoms (e.g. wheezing)- 1.4%. Symptomatic hypotension and bradycardia were rare occurrences (less than 1%). [8,9]

While sleep disturbances are common during infancy, nightmares are extremely unusual, suggestive that, at least in a small number of patients, this is the direct effect of the medication on the central nervous system.
To date, there is substantial animal and human evidence that the endogenous β-adrenergic system is essential for communication between amygdala and hippocampus, modulating the strength of memories, particularly those associated with emotional arousal or traumatic events. [10] Because of its ability to cross the blood brain barrier and block the β-adrenergic system, propranolol has emerged as a therapy for post-traumatic stress disorder and drug addiction. When given to healthy volunteers, propranolol led to impairment of working and emotional memory, while patients treated for hypertension experienced impairment of verbal memory.[11]
The effects of prolonged suppression of the adrenergic system in children are not currently known. However, there is evidence that structural damage of the hippocampal region in infant monkeys led to severe memory loss, social interaction deficits and locomotor stereotypia. [12] Similarly, infants with isolated hippocampal injury, documented on MRI, due to hypoxic ischemic encephalopathy had evidence of severe impairment of episodic memory. More significantly, these deficits were not apparent until 5-6 years of age.[13]
Therefore, animal, human and indirect structural data all raise a legitimate concern about the long-term impact of chronic propranolol use on patients treated for IH during the most susceptible developmental period.

Due to its potential short and long-term effects on the developing brain, we have explored the use of other non-selective beta-blockers. Nadolol is a synthetic, non-selective beta-blocker with no intrinsic sympathomimetic or membrane stabilizing activity.[14] It is currently being used in the pediatric age group for hypertension, rhythm abnormalities (atrial tachyarrhythmias, ventricular arrhythmias) and treatment of overt aggression in developmentally delayed individuals. [15]The usual dose is 1-4 mg/kg/day orally and it has a 1:1 equivalence dose ratio

with propranolol. Unlike propranolol, nadolol is not metabolized by the liver, and is excreted unchanged, primarily by the kidneys. The half-life of nadolol is 12 – 24 hrs, reaching steady state serum concentrations in 6 to 9 days with once daily dosage. [14] Nadolol has no intrinsic sympathomimetic activity, and in contrast to propranolol, has little myocardial depressant activity and does not have an anesthetic like membrane stabilizing action.[16] In addition, Nadolol does not cross the blood brain barrier, and therefore has less potential for CNS adverse events.

The potential side effects of nadolol are similar to propranolol: bradycardia, hypotension, rhythm/conduction disturbances (first degree and third degree heart blocks), hypoglycemia, fatigue, bronchospasm, hypothermia, etc. (16) However, the incidence of side effects in two nadolol trials was so low that statistical analysis of individual side effects could not be conducted.[15,17] The reported adverse effects were not dose dependent.

We undertook a pilot study (funded by PSI) to examine the efficacy and relative safety of nadolol through a comparison with a historical cohort treated with propranolol. At similar mean dosages (2.1 mg/kg/day), patients receiving nadolol (n=10) had a mean percentage IH involution of 51 ± 18.45% at the 4-week visit, 83 ± 13.86% at 12-week visit and **97 ± 3.05%** at the end of the study (24 weeks). In contrast, propranolol patients (n=10) had a less favourable response: 28 ± 10.44%, 56 ± 16.55% and **86 ± 14.82%** at 4, 12 and 24 weeks, respectively (p <0.001). [18] Nadolol was well tolerated with no significant adverse events.

These preliminary data supports our hypothesis that nadolol is an efficacious and given its pharmacological properties, potentially safer alternative to propranolol for use in the treatment of IH.

References:

1. Drolet BA, Esterly NB, Frieden IJ. Hemangiomas in children. N Engl J Med 1999;341:173-81.
2. Metry DW, Hebert AA. Benign cutaneous vascular tumors of infancy: when to worry, what to do. Arch Dermatol 2000;136:905-14.
3. Frieden IJ, Haggstrom AN, Drolet BA, et al. Infantile hemangiomas: current knowledge, future directions. Proceedings of a research workshop on infantile hemangiomas, April 7-9, 2005, Bethesda, Maryland, USA. Pediatr Dermatol 2005;22:383-406.
4. Leaute-Labreze C, Dumas de la Roque E, Hubiche T, Boralevi F, Thambo JB, Taieb A. Propranolol for severe hemangiomas of infancy. N Engl J Med 2008;358:2649-51.
5. Wood AJ, Carr K, Vestal RE, Belcher S, Wilkinson GR, Shand DG. Direct measurement of propranolol bioavailability during accumulation to steady-state. Br J Clin Pharmacol 1978;6:345-50.
6. Neil-Dwyer G, Bartlett J, McAinsh J, Cruickshank JM. Beta-adrenoceptor blockers and the blood-brian barrier. Br J Clin Pharmacol 1981;11:549-53.
7. Storch CH, Hoeger PH. Propranolol for infantile haemangiomas: insights into the molecular mechanisms of action. Br J Dermatol 2010;163:269-74.
8. Marqueling AL, Oza V, Frieden IJ, Puttgen KB. Propranolol and infantile hemangiomas four years later: a systematic review. Pediatr Dermatol 2013;30:182-91.
9. Drolet BA, Frommelt PC, Chamlin SL, et al. Initiation and use of propranolol for infantile hemangioma: report of a consensus conference. Pediatrics 2013;131:128-40.

10. McGaugh JL. The amygdala modulates the consolidation of memories of emotionally arousing experiences. Annu Rev Neurosci 2004;27:1-28.
11. McAinsh J, Cruickshank JM. Beta-blockers and central nervous system side effects. Pharmacol Ther 1990;46:163-97.
12. Bachevalier J, Alvarado MC, Malkova L. Memory and socioemotional behavior in monkeys after hippocampal damage incurred in infancy or in adulthood. Biol Psychiatry 1999;46:329-39.
13. Gadian DG, Aicardi J, Watkins KE, Porter DA, Mishkin M, Vargha-Khadem F. Developmental amnesia associated with early hypoxic-ischaemic injury. Brain 2000;123 Pt 3:499-507.
14. Helfand M, Peterson K, Dana T. In: Drug Class Review on Beta Adrenergic Blockers: Final Report. Portland (OR); 2007.
15. Connor DF, Ozbayrak KR, Benjamin S, Ma Y, Fletcher KE. A pilot study of nadolol for overt aggression in developmentally delayed individuals. J Am Acad Child Adolesc Psychiatry 1997;36:826-34.
16. Dreyfuss J, Brannick LJ, Vukovich RA, Shaw JM, Willard DA. Metabolic studies in patients with nadolol: oral and intravenous administration. J Clin Pharmacol 1977;17:300-7.
17. Mehta AV, Chidambaram B. Efficacy and safety of intravenous and oral nadolol for supraventricular tachycardia in children. J Am Coll Cardiol 1992;19:630-5.
18. Pope E, Chakkittakandiyil A, Lara-Corrales I, Maki E, Weinstein M. Expanding the therapeutic repertoire of infantile haemangiomas: cohort-blinded study of oral nadolol compared with propranolol. Br J Dermatol 2013;168:222-4.

Objectives

To compare the efficacy and safety of dose equivalent of oral propranolol versus oral nadolol in patients with infantile hemangiomas

Methods

A: Participants, interventions, outcomes

A1. Study Settings

The study will be conducted at the Dermatology Clinic, the Hospital for Sick Children, Toronto, a tertiary academic centre with a province wide referral base.

A2. Eligibility Criteria

Inclusion criteria:

- 1-6 months corrected age
- Written parental informed consent
- At least one of the following:
  - Size: hemangioma >1.5 cm on the face or >3 cm on other body parts
  - Causing or with potential for functional impairment (e.g. amblyogenic IH, ulcerated hemangioma)
  - Causing or with potential for cosmetic disfigurement (e.g. nasal tip, glabella location)

Exclusion criteria:

- Contraindications to beta-blockers
  - Hypotension
  - Bradycardia
  - Hypoglycemia
  - Cardiac disease associated with decreased ejection fraction and/or ≥ second degree heart block
  - Bronchospasm (including bronchial asthma)
  - Allergic rhinitis
- Corrected gestational age less than 1 month at screening
- Patients with PHACES cerebral arteriopathy at risk of stroke
- Patients and/or breastfeeding mothers receiving treatment with anti-arrhythmic agents, calcium channel blockers, ACE inhibitors, inotropic agents, vasodilators, hypoglycemic agents, neuroleptics, antiacids, benzodiazepines, thyroxine, warfarin
- Patients treated with an oral beta-blocker or other agent (e.g. systemic steroids, vincristine) within 2 weeks from randomization
- Patients treated with topical timolol within 1 week from randomization
- Vascular tumors other than infantile hemangioma (e.g. pyogenic granuloma, hemangioendothelioma)

A3. Interventions

Subjects will be randomized to either nadolol or propranolol.

Nadolol: oral suspension, 10 mg/ml. The suspension will be compounded by Sickkids pharmacy and provided in OralMix vehicle, which provide medication with expiry time up to 91 days after preparation (at room temperature).

Propranolol: oral suspension, 5 mg/ml. The suspension will be prepared at Sickkids pharmacy and suspended in Oral-Blend-SF* (sugar-free) vehicle. Storage – up to 91 days after preparation (at room temperature: 15-30*C).

Dosage: Irrespective of intervention, patients will be administered twice-daily doses. From visit 1-3, patients will be given incremental doses of medication as follows: Day 0- 0.66 mg/kg/day divided BID; Day 7-1.3 mg/kg/day divided BID and Day 14- 2 mg/kg/day divided BID. In all subsequent visits the dosage will be adjusted based on the current weight rather than the baseline weight to maintain 2 mg/kg/day until Week 24. The dose may be escalated to 3 mg/kg/day if at any point before 24 weeks the investigators assess the need for a higher dose. After Week 24 (end of the study) investigators will follow the procedure outlined in **Figure 1**.

The dose calculated for the study patients will be rounded up or down to the nearest 0.5 mg (which is nearest measurable 0.1 mL of propranolol and 0.05 mL for nadolol).

The families will be provided with the following guidelines regarding missing dose/vomiting:

- Vomiting within 15 min from the administrated dose – repeat the same dosage;
- Vomiting > 15 min from the administrated dose – DO NOT readminister the dose;
- Missed dose within- the 6 hours from the from the last dose – administer the drug;
- Missed dose < 6 hours from the next dose – wait until the next scheduled dose.

Figure 1: Dosage procedure

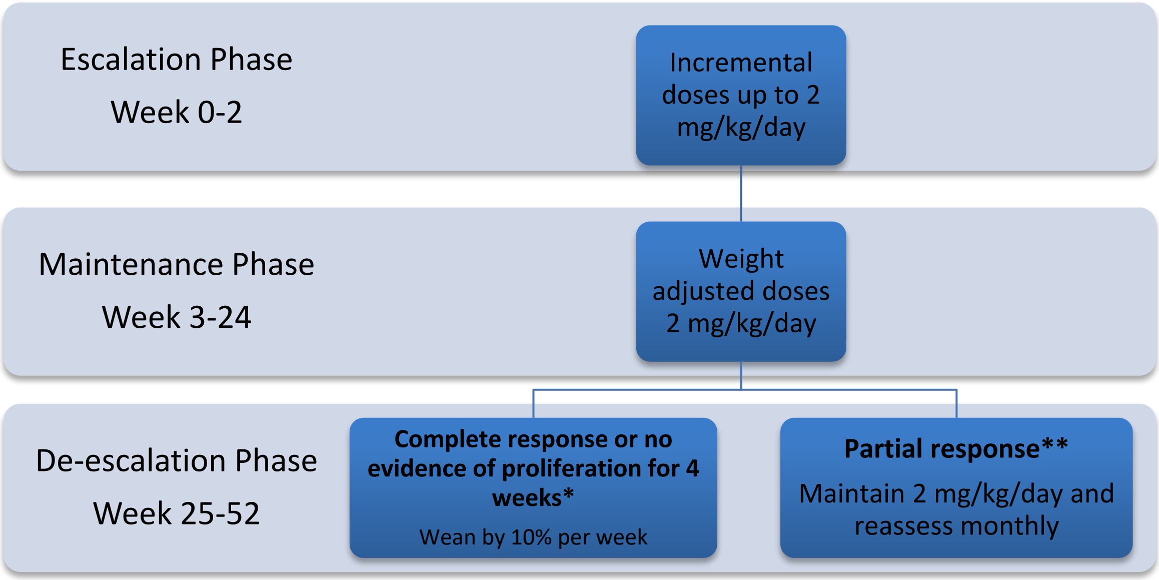

Legend:
*- Complete response is defined as complete involution or minimal residual epidermal changes (telangiectasias, anetoderma, gray-red discoloration)
**- Partial response is defined by decrease in the size, extent and coloration of the IH but with persistent redness, bulk and increased local temperature that in the opinion of the treating physician would benefit from further therapy.

A4. Procedure

Overall Schedule of Events (Appendix 1)

Patients will be identified as potential subjects during their assessment in the Dermatology Clinic. If they meet eligibility criteria and parents sign the consent form, their name will be submitted to the research pharmacy for randomization.
Whenever possible, the baseline visit may occur the same day as the screening visit. At Day 0, patient will be dispensed the medication. Vitals signs (blood pressure, heart rate, respiratory rate and temperature) will be obtained and patient will receive the first dose of the intervention in clinic. The procedure will be repeated with each dose escalation (Day 0, Day 7, Day 14). After approximately 1 hour, blood sugar levels will be checked via pinprick and vital signs will be obtained. The following guidelines are recommended for abnormal findings:

i. Asymptomatic patient with blood sugar < 2.5 mmol/L (45mg/dL) and/or a drop in BP and HR of more than 20% from baseline or less than 5%- ile for age
    a. Hourly monitoring for 3 hours

   b. Normalization of findings, patient is sent home on no medication and brought back the within 1-7 days for a second dose trial
   c. Persistent abnormalities or reoccurrence of the findings with second dose challenge, patient undergoes early study termination

ii. <u>Symptomatic</u> patient with blood sugar < 2.5 mmol/L (45mg/dL) or a drop in BP and HR of more than 20% from baseline or less than 5%-ile for age
   a. Management of abnormalities including admission if indicated
   b. Early study discontinuation

Growth Parameters and Vital Signs

Growth parameters (height- Ht and weight- Wt) and vital signs consisting of heart rate (HR), blood pressure (BP), respiratory rate (RR) and axillary temperature (T) will be obtained at each visit. Additionally, approximately 1 hour after each dose escalation (D0, W1 and W2) patients will have their BP, HR measured. Bradycardia will be defined as a drop of >20% from baseline value or less than 5%ile for age (1-12 months <80 bpm; ≥12 months < 70 bpm). Hypotension will be defined as a drop of more than 20% from baseline or less than 5%ile oscillometric for age (1-6 months < 85 mmHg; 6-12 months < 88 mmHg)[9]

Medication Review

A complete medication review before enrolment that includes both prescribed and over the counter preparation will be conducted. Additionally, immunization history, allergy history and current medications of breastfeeding mothers will be elicited.

Medical assessment

Medical assessment will consist of a full skin and systems examination with focus on the evaluation of skin lesions, respiratory, cardiovascular and abdominal structures (Appendix 3: Data Collection Form). Follow-up visits will also include a physician assessment of IH's progression.

Clinical photographs

At least 2 digital photographs will be taken of the target IH (in patients with more than one IH meeting the inclusion criteria, the target lesion will be at the discretion of the family). The first photograph will be taken parallel with the IH (front-on view) and the second at the angle so that the thickness of the IH can be clearly visualized (90* from front-on view position -side-on-view) (Appendix 2: Standardized operating procedure for photography taking). The photograph will be labeled with the patient's ID and the visit number. At the end of the study, one independent, external, blinded assessor, will score the change in the bulk and other characteristics of IH by comparing photos at each visit with the baseline ones.

A5. Outcomes

Primary outcome measure:

IH are 3D lesions with variable extent and depth of involvement (superficial, deep or mixed) and color. As such, it is difficult to adequately quantify the size of one particular lesion and to monitor proliferation or involution over time. Furthermore, they occur at a time when human body grows very rapidly. These factors limit a universal, standardized way of measuring IH over time/with treatment and to date there are no validated tools. A visual analog scale (VAS) was modified and used by us for several other studies on IH as a way of quantifying change in the visible bulk (size/extent) and color of the lesion. This consists of a 100 mm scale with 3 anchors (-100= ongoing proliferation, 0=no change, +100= complete shrinkage of the tumor), where 5mm represents 10% change in either direction. The change in bulk is assessed by comparing clinical photographs at various time points. This version of the VAS has been used

in several other studies, showing great inter-person reliability (ICCs: 0.87, p<0.001) and responsiveness to change. [18-20]

The primary efficacy criterion is the change in the bulk (size/extent) and color of the IH at Week 24 compared to baseline using VAS, calculated by averaging the VAS for size/extent and VAS for color. This will be independently assessed by one external assessor and the treating investigator, by comparing standardized photographs at Week 24 and baseline.

Secondary outcome measures:

1. Efficacy secondary outcome measures
    a. Percent change in IH bulk using VAS at 4, 12, 52 weeks
    b. Percentage of patients achieving 75% and 100% involution using VAS at 24 and 52 weeks
    c. Inter-rater reliability of the VAS scores
    d. Percentage of patients achieving functional correction at 4, 12, 24 and 52 weeks (e.g.
    e. Time to achieve 75% and 100% tumor shrinkage
    f. Percent change in the volumetric changes of hemangioma using the formula $[(\text{Length} + \text{Width})/2]^3 \times 0.07$ at 24 and 52 weeks [21]
    g. Percentage of patients with residual changes (telangiectasias, discoloration, fibro-fatty changes, anetoderma); for the purpose of the analysis the following grouping will be used
        - telangiectasias
            1. mild- fine telangiectasias covering less than 10% of the lesion
            2. moderate- telangiectasia covering 11- 49% of the lesion
            3. severe- coarse telangiectasia covering >50% of the lesion
        - discoloration
            1. mild- grayish discoloration
            2. moderate- violaceous discoloration
            3. severe- deep red discoloration
        - fibro-fatty changes
            1. mild- small elevation above surface
            2. moderate- moderate elevation
            3. severe- marked elevation and/or distortion of the landmarks
        - anetoderma (atrophy, loss of skin elasticity)
            1. mild- fine wrinkling of the skin
            2. moderate- wrinkling of the skin causing minimal sagging of the skin
            3. severe- wrinkling of the skin and excessive loose skin
2. Safety outcome measures
    a. Frequency of observed and reported adverse events (documented in parent diary)

A6. Target sample size

We estimate that 40 patients are needed in each group. This will ensure that the study will have 84% power to determine that nadolol is <u>non-inferior</u> to propranolol at an <u>non-inferior</u> margin of 10%, a significance level of 2.5%, based on the assumption that standard deviation is 15% for each of the groups (as seen in our pilot study). [18]

A7. Recruitment and Retention

Patients will be recruited during their regular visits to the Dermatology Clinic. Additionally, the study will be advertised through the Toronto paediatric and dermatology newsletters. We expect that we will enroll the number needed within 9 months given the referral base. In our experience, parents of patients with IH are highly motivated to participate in clinical studies that will offer a benefit to their infants. We expect less than 1% withdrawals from the study.

A8. Study Timeline

The study duration will be 24 weeks; however, patients will be followed up to 52 weeks. Based on the referral patterns for the past 5 years, we expect that all patients will be enrolled within the first 9 months.

Figure 2: Study Timeline

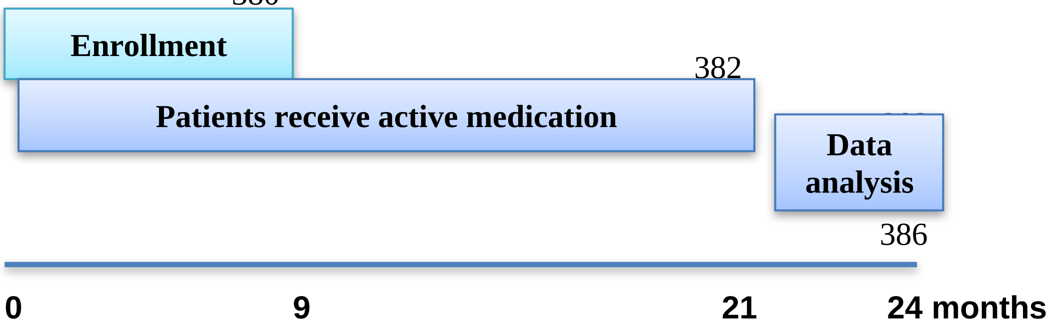

B: Assignment of Interventions

B1. Randomization

A block of 4 randomization schedule with equal allocation to both groups will be prepared by the research pharmacist and kept securely with the SickKids Research Support Pharmacy. The pharmacist will be unblinded to the intervention.

B2. Blinding

Parents and treating investigators will be blinded to the intervention until Week 24 visit. Unmasking during this period will be allowed only in cases of serious adverse events. Principal investigator will provide verbal instruction to the pharmacist requesting preliminary unblinding of the study participant. As the potential adverse events are similar for both drugs, it is unlikely that unblinding is necessary to provide treatment.

At week 24 the study investigator and the family will be unblinded. The study participants will continue to take the same medication (at the same dose or lower depending on the hemangioma's response). This medication will not be provided by the Research Supporting Pharmacy.

Each study participant will continue to be followed by the study doctor at a frequency dictated by the clinical progress, but only three more visits to SickKids/Dermatology will be considered as the study visits: Week 32, 40, and 52 (end of the study participation).

One of the Dermatology clinic nurses (the study team member) will be unblinded in order to provide counseling to the study patients in terms of dose/volume of drug.

C: Data Collection, management and analysis

C1. Data collection and management methods

Data collection will be performed at each time points as outlined in Appendix 1. An assessor blinded to the intervention will perform scoring of outcome measures at the end of the study. Data storing and analysis will be facilitated using Redcap database.

C2. Statistical Methods

An intention to treat analysis (ITT) will be conducted. Descriptive statistics (means and standard deviation for continuous data and frequencies and percentages for categorical data) will be used to summarize the data. A two sample independent t-test will be used to test the difference of percentage change from baseline to week 24 between propranolol and nadolol groups. The non-inferior margin is assumed to be 10%. A 95% CI interval of the difference will be calculated. If the lower bound of the 95 %CI is bigger than the non-inferior margin, we will conclude that nadolol is non-inferior to propranolol.

Secondary analyses will be conducted for week 4, 12, 24, and 52, using a mixed model analysis. This model will assess the difference in the tumor characteristics between the two groups. The interaction in the model will represent the difference between two groups. A best correlation matrix will be estimated and used to account for the correlation within the subjects.

For time to achieve 75% and 100% tumor shrinkage, survival analysis will be performed. Kaplan-Meier curves will be used to estimate the time to 75% and 100% tumor shrinkage. Log rank tests will be used to compare the differences in the curves. Cox proportional hazard model will be used to compare the hazard ratio of between the groups adjusted for location (facial vs. non-facial), age and other clinically deemed variables.

References

18. Pope E, Chakkittakandiyil A, Lara-Corrales I, Maki E, Weinstein M. Expanding the therapeutic repertoire of infantile haemangiomas: cohort-blinded study of oral nadolol compared with propranolol. Br J Dermatol 2013;168:222-4.

19. Pope E, Doria AS, Theriault M, Mohanta A, Laxer RM. Topical imiquimod 5% cream for pediatric plaque morphea: a prospective, multiple-baseline, open-label pilot study. Dermatology 2011;223:363-9.

20. Pope E, Krafchik BR, Macarthur C, et al. Oral versus high-dose pulse corticosteroids for problematic infantile hemangiomas: a randomized, controlled trial. Pediatrics 2007;119:e1239-47.

21. Tsang MW, Garzon MC, Frieden IJ. How to measure a growing hemangioma and assess response to therapy. Pediatr Dermatol 2006;23:187-90.

4. Appendices

APPENDIX 1: Schedule of events

	Screen	V1<br>Day 0<br>Baseline	V2<br>Day<br>7+/-3	V3<br>Day<br>14+/-3	V4<br>Day<br>21+/-3	V5-V13<br>Week<br>4,8,12,16,20,24<br>(+/- 3 days)<br>,32,40,52<br>(+/-7days)
**Review inclusion/ exclusion criteria**	X					
**Consent**	X					
**Height, weight, blood pressure, heart rate, respiratory rate, axillary temperature**	X	X	X	X	X	X
**Medication review**	X	X	X	X	X	X
**ECG***	X					
**Medical assessment**	X	X	X	X	X	X
**Clinical photographs**		X	X	X	X	X
**Adverse events review**			X	X	X	X
**Medication administration**		X	X	X	X	
**Blood sugar (pin prick) and blood pressure, heart rate ^**		X	X	X	X	

*- single lead ECG performed before medication start; abnormal findings may require an echocardiogram at the discretion of the local cardiologist

^- at 1 hour post dose escalation; a drop of >20% from baseline or values less than 5%-ile for age and a blood sugar of <2.5 mmol/L (45mg/dL) will be considered abnormal

APPENDIX 2

Standard operation procedures for photography

Serial photography – Digital camera

Aim - control over all photographic variables. The only observable change from one time point to the next should reflect a change in the lesion(s) of interest.

Variables:

- patient preparation
- patient positioning
- camera to subject registration
- reproduction ratio (magnification)
- lighting & exposure
- preparation of digital camera

Photography acquisition:

1. Patient preparation:
   Patient undressed appropriate to the target areas to be photographed. The parents' chest will be considered as a background. At each patient visit the parent will wear a plain light blue non-reflective closes in order to avoid reflections that might compromise image quality.
2. Patient positioning (refer to photos from baseline visit)
   The baby parents will be sitting on a chair with the baby sitting on his/her knees, if possible, or in his/her arm. At baseline, the dermatologist will analyze the best angle at which each photograph should be taken. 2 Photographs will be acquired at 2 angles defined at baseline:
   1. **Front-on-view of IH**: photo of target IH taken at the angle so that the image plane is parallel to the Region of Interest (ROI) at baseline - biggest diameter of IH.
   2. **Side-on-view of IH**: photo of target IH taken at the angle defined at baseline so that the thickness of the lesion can be clearly visualized.

   For a given patient, photograph 1 and 2 have to be acquired at the same angle as at baseline for all subsequent visits.

3. Camera to subject registration

   In order to standardize the distance and approach angles between the camera and the patient, a position indicator will be used for the study. It will contain pre-printed marks for the positioning of the baby's parents chair and the tripod where the camera will be placed. The position indicator is a square shaped plastic carpet with a pre-printed circle of fixed diameter (d=100cm). The circle radius is marked every 15 degree. Fix the camera to a tripod.

4. Reproduction ratio (magnification)

   Take all the study pictures with maximum zoom on camera.

5. Lighting & exposure

   Same room location will be used for pictures acquisition. Attach the system flash to the camera and turn on.

6. ID labels and Munsell colour chart

   For each photograph acquisition, the patient ID label with Munsell colour chart will be held by the parent parallel to the camera but without covering any part of the Region of Interest (IH).

   The Munsell colour chart contains a checkerboard array of 24 coloured squares with known colorimetric properties that are scientifically designed to help determine the true clolour balance of any colour rendition system.

7. Preparation of digital camera and ID card with Munsell chart holder

   Camera on, flash on. Enter the information on the photograph ID card (should include subject number, DOB, date of picture acquisition, study visit number). Put the Munsell colour chart in chart holder with subject data.

   Take 2 picture of each view of target area.

APPENDIX 3
Monitoring and Addressing Adverse Events

Definition: An Adverse Event (AE) is any undesirable experience associated with the use of any of the two interventions

Grading AE:
- mild: awareness of signs and symptoms that are easily tolerated
- moderate: signs and/or symptoms that interfere with usual activities
- severe: signs and/or symptoms that cause inability to pursue usual activities

Serious AE:
Events that lead to any of the following:
- death
- life-threatening
- initial or prolonged hospitalization
- disability or permanent damage
- congenital defect
- require intervention to prevent permanent damage
- may require other intervention to prevent one of the outcomes listed above

Monitoring for adverse events:
- *during clinic visits*
    - protocol for dose escalation with monitoring of vital signs and laboratory investigations and early termination if necessary (see procedures)
    - physical examination
    - review of the parental diaries
- *home monitoring* (parental diary)
    - parents are instructed to record any unusual occurrences or changes in the usual functioning of the child; these refer, but are not limited, to the following:
        - illnesses
        - difficulty to arouse
        - pallor
        - difficulty feeding
        - respiratory difficulties
        - decreased level of activity
        - mood changes
        - behavioral changes
        - nightmares and other sleeping difficulties

Medication discontinuation /Early termination
The intervention will be discontinued and patient will exit the study in the following circumstances:
- moderate to severe AE in the opinion of the investigator and/or parent (e.g respiratory difficulties that do not resolve with medication use, sleeping difficulties that affect the functionality of the child, extreme behavioural and/or personality changes, etc.)
- any serious AE irrespective of the severity
- persistent or re-occurrence of bradycardia and /or hypotension/ or hypoglycemia.

Reporting of **Adverse Events**

All adverse events will be reported to the Hospital for Sick Children Research Ethics Board according to the Hospital for Sick Children's adverse event reporting requirements. All serious, unexpected adverse drug reactions to the study medication will be reported to Health Canada within 15 calendar days or for death or life-threatening events, within 7 calendar days. In the latter case, a follow-up report must be filed within 8 calendar days. Adverse reactions will be managed according to the Hospital for Sick Children's standard clinical management practices.
