# Supplementary material for: Noninferiority and Safety of Nadolol vs Propranolol in Infants With Infantile Hemangioma: A Randomized Clinical Trial
Source: JAMA Pediatr. 2021 Nov 8;176(1):1–8. doi: 10.1001/jamapediatrics.2021.4565 (PMC8576629; doi:10.1001/jamapediatrics.2021.4565)
Supplement: Supplement 2. — eTable. Secondary efficacy outcome measures eFigure 1. Efficacy data boxplots eFigure 2. Visual correction of IH color chart [file jamapediatr-e214565-s002.pdf]

Supplemental Online Content

Pope E, Lara-Corrales I, Sibbald C, Liy-Wong C, Kanigsberg N, Drolet B. Noninferiority and safety of nadolol vs propranolol in children with infantile hemangioma: a randomized clinical trial. *JAMA Pediatr*. Published online November 8, 2021.
doi:10.1001/jamapediatrics.2021.4565

eTable. Secondary efficacy outcome measures

eFigure 1. Efficacy data boxplots

eFigure 2. Visual correction of IH color chart

This supplemental material has been provided by the authors to give readers additional information about their work.

© 2021 Pope E. et al. *JAMA Pediatrics.*

eTable. Efficacy data: secondary efficacy outcome measures

Characteristic(s)	Nadolol N1=35	Propranolol N2=36	P value	Nadolol N1=38	Propranolol N2=31	P value
Period	***0-24 weeks***			***24-52 weeks***		
VAS size shrinkage, mean% (SD)	97.9 (3.7)	89.1 (17.7)	**0.005**	99.6 (1.1)	93.6 (10.9)	**0.001**
VAS color fading, mean% (SD)	94.5 (8.7)	80.5 (17.9)	**<0.001**	97.3 (3.8)	87.2(16.1)	**0.001**
VAS average (extent and color fading), mean% (SD)	96.4 (5.7)	86.8 (16.6)	**0.002**	98.6 (2.2)	91.9 (12.7)	**0.002**
75% tumor shrinkage, n (SD)	35 (100)	31 (86.1)	0.068	38 (100)	29 (93.5)	0.386
100 % tumor shrinkage, n (SD)	16 (45.7)	10 (27.8)	0.186	32 (84.2)	16 (51.6)	**0.008**
HAS, mean (SD)	1.60 (1.7)	3.20 (2.3)	**0.002**	0.6 (0.8)	1.56 (1.4)	**0.001**
Residual changes, n (%)						
- Telangiectasias	18 (54.4)	12 (35.3)	0.181	21 (55.3)	10 (41.7)	0.434
- Discoloration	14 (42.4)	23 (67.6)	0.067	14 (36.8)	12 (50)	0.448
- Fibrofatty	17 (51.5)	24 (70.6)	0.177	7 (18.4)	8 (33.3)	0.303
- Anetoderma	9 (27.3)	4 (11.8)	0.195	8 (21.1)	2 (8.3)	0.331
Functional involution[#], n/N affected	2/5	3/6	1.000	0/7	2/6	NA

© 2021 Pope E. et al. *JAMA Pediatrics.*

eFigure 1. Efficacy data boxplots

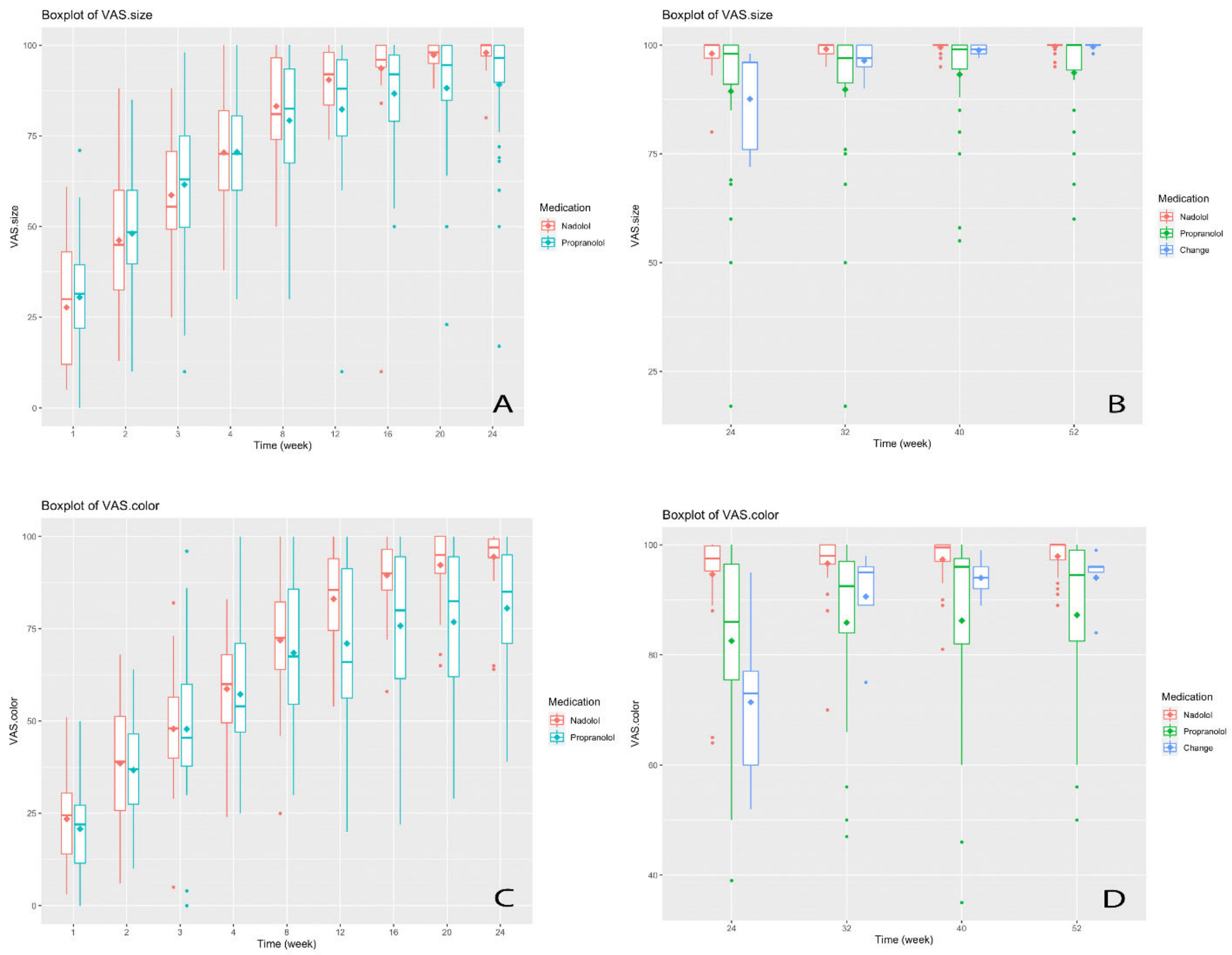

Boxplots with percentage response for size and color compared to baseline. Panels A (0-24 weeks) and B (24-52) refer to size involution percentages with “change” group representing people who changed from propranolol to nadolol after week 24. Panels C (0-24 weeks) and D (24-52 weeks) show color improvement from baseline.

© 2021 Pope E. et al. *JAMA Pediatrics.*

eFigure 2.: Visual correction of IH color chart

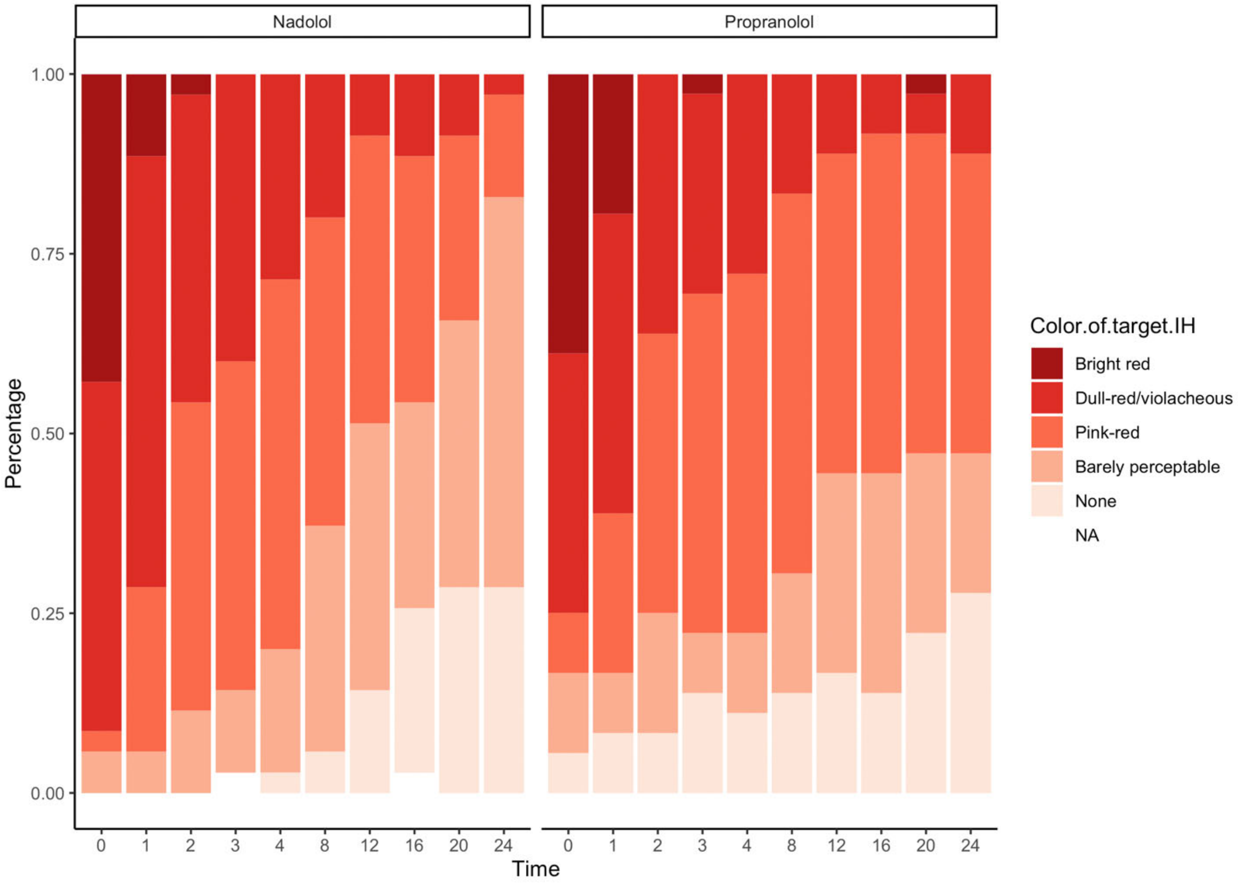

© 2021 Pope E. et al. *JAMA Pediatrics.*
