# Supplementary material for: Noninferiority and Safety of Nadolol vs Propranolol in Infants With Infantile Hemangioma: A Randomized Clinical Trial
Source: JAMA Pediatr. 2021 Nov 8;176(1):1–8. doi: 10.1001/jamapediatrics.2021.4565 (PMC8576629; doi:10.1001/jamapediatrics.2021.4565)
Supplement: Supplement 3. — Data sharing statement [file jamapediatr-e214565-s003.pdf]

Data Sharing Statement

Pope. Noninferiority and Safety of Nadolol vs Propranolol in Infants With Infantile Hemangioma. *JAMA Pediatr*. Published November 08, 2021. doi:10.1001/jamapediatrics.2021.4565

Data
Data available: No

Additional Information
Explanation for why data not available: we are happy to share aggregated, un-identified data
